# Identifying SARS-CoV-2 Drugs Binding to the Spike Fatty Acid Binding Pocket Using In Silico Docking and Molecular Dynamics

**DOI:** 10.3390/ijms24044192

**Published:** 2023-02-20

**Authors:** Sakshi Piplani, Puneet Singh, Nikolai Petrovsky, David A. Winkler

**Affiliations:** 1 College of Medicine and Public Health, Flinders University, Bedford Park 5046, Australia; 2 Vaxine Pty Ltd., 11 Walkley Avenue, Warradale 5046, Australia; 3 Department of Biochemistry and Chemistry, La Trobe Institute for Molecular Science, La Trobe University, Melbourne 3086, Australia; 4 Monash Institute of Pharmaceutical Sciences, Monash University, Parkville 3052, Australia; 5 School of Pharmacy, University of Nottingham, Nottingham NG7 2RD, UK

**Keywords:** SARS-CoV-2, fatty acid binding pocket, drug repurposing, molecular docking, molecular dynamics

## Abstract

Drugs against novel targets are needed to treat COVID-19 patients, especially as SARS-CoV-2 is capable of rapid mutation. Structure-based de novo drug design and repurposing of drugs and natural products is a rational approach to discovering potentially effective therapies. These in silico simulations can quickly identify existing drugs with known safety profiles that can be repurposed for COVID-19 treatment. Here, we employ the newly identified spike protein free fatty acid binding pocket structure to identify repurposing candidates as potential SARS-CoV-2 therapies. Using a validated docking and molecular dynamics protocol effective at identifying repurposing candidates inhibiting other SARS-CoV-2 molecular targets, this study provides novel insights into the SARS-CoV-2 spike protein and its potential regulation by endogenous hormones and drugs. Some of the predicted repurposing candidates have already been demonstrated experimentally to inhibit SARS-CoV-2 activity, but most of the candidate drugs have yet to be tested for activity against the virus. We also elucidated a rationale for the effects of steroid and sex hormones and some vitamins on SARS-CoV-2 infection and COVID-19 recovery.

## 1. Introduction

The COVID-19 pandemic has impacted health systems around the world. Over 80% of the global population has been infected, and millions have died. New drugs are needed to treat SARS-CoV-2 as the virus mutates often to escape therapeutic monoclonal antibodies. In silico simulations can rapidly identify putative drugs against SARS-CoV-2. Repurposing existing drugs can provide SARS-CoV-2 drugs with known safety profiles [1]. Few drugs are approved for emergency use.

**Figure 1:**
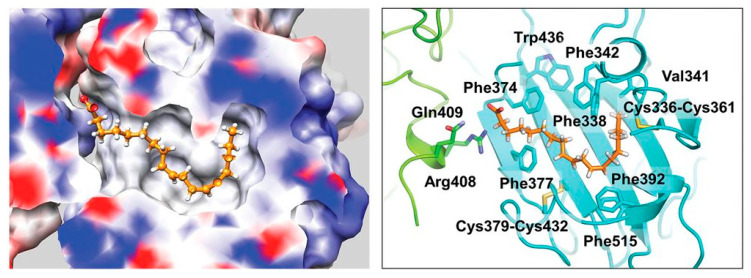
FABP with linoleic acid bound (**left**) and residues lining the pocket (**right**). CC BY 4.0 licence from Toelzer et al. [3].

The envelope spike (S) protein plays a crucial role in coronavirus infection and pathogenesis [2]. SARS-CoV-2 has a highly glycosylated S protein that belongs to the class of trimeric class I viral fusion glycoproteins. The S protein is 1273 amino acid long and comprises three major subunits, S1, S2, and S2′. These subunits undergo conformational changes during virus-host membrane fusion. Toelzer et al. discovered that a free fatty acid (FFA), linoleic acid (LA), binds to a hydrophobic pocket in the S protein adjacent to the receptor binding domain (RBD) [3]. LA is an essential omega-6 poly-unsaturated fatty acid (PUFA). LA binding to S protein stabilizes the compact, closed conformation that blocks binding to human angiotensin converting enzyme 2 (ACE2), the target of the virus. FFA-binding pockets (FABPs) in proteins are “greasy” tubes lined by hydrophobic amino acids that accommodate the hydrocarbon tail, and a hydrophilic positively charged anchor for the acidic headgroup of the FFA. The S protein pocket is a bent tube formed from phenylalanines and the anchor provided by Arg408 and Gln409 from the adjacent RBD in the trimer. LA binds to all three binding pockets in the S trimer in the unsymmetric (C1) closed structure. Glycosylation sites are far from the FABP and are unlikely to affect LA binding. In human cells, LA acts synergistically with remdesivir to inhibit SARS-CoV-2 [3]. The FABP is also present in SARS and MERS coronavirus S proteins [3]. In the SARS, the FABP is flanked by a gating helix, as in SARS-CoV-2, with Arg395 and Gln396 of SARS-CoV positioned 10 and 11 Å from the entrance. Its conformation is virtually identical to that of apo SARS-CoV-2 (Figure 1). Polyunsaturated fatty acids such as linolenic acid and eicosapentaenoic acid also inhibit SARS-CoV-2 spike protein pseudo-virion binding and entry to A549/hACE2 cells in vitro [4].

Given the presence of a FABP in SARS and SARS-CoV-2 S proteins, structure-based de novo drug design and repurposing of drugs and natural products is a rational approach to discovering effective therapies. Several computational studies have reported compounds that inhibit binding of the S protein RBD to human ACE2, e.g., [5]. However, screening for agents that bind the FABP and stabilize the closed conformation required to inhibit binding to ACE2 is a viable alternative. For example, Shoemark et al. reported a docking and molecular dynamics (MD) study of binding of LA and other ligands to the FABP. This suggested linoleate and dexamethasone stabilized the locked S conformation, while cholesterol destabilized it by binding to another site in the hinge region [6]. Another study of eight putative binding sites on the S protein identified additional ligands that bind to the FABP and stabilize the closed conformation [7].

We have reported computational studies on S protein and ACE2 to elucidate the origins of SARS-CoV-2 and identify repurposed drugs inhibiting its main protease, RNA-dependent RNA polymerase, and helicase [8,9,10,11]. Here, we used the S protein FABP structure to identify repurposing candidates as SARS-CoV-2 treatments. This binding pocket has not yet been extensively studied from a therapeutic standpoint, and it offers a novel way of controlling the folding of the spike to inhibit its binding to the target receptor, ACE2. We also extended previous analyses on the FABP in the wildtype Wuhan-Hu-1 virus strain to include the more recent Omicron variants.

## 2. Results

We established molecular docking and molecular dynamics protocols for predicting the affinity of the SARS-CoV-2 spike for ACE2 from different species and small drug repurposing candidates for key protein targets in the virus. These protocols identified potential drug repurposing candidates, >30% of which were found to be activity against the virus or relevant protein target. This protocol (see Methods) was used to identify binding to the spike FABP.

The binding energies of the top 100 repurposing drug candidates selected from the docking scores and subjected to MD simulations are summarized in Table 1. Most compounds are extended lipophilic molecules with logP (octanol/water) values between 4 and 7 (Table S1). They belong to diverse pharmacological classes, their similarities being extended shapes, high lipophilicity, and terminal functional groups (often carboxylic or hydrogen bond donor or acceptor) able to interact with the anchor residues. A substantial number of drugs in Table 1 have been reported by other computational studies to inhibit other SARS-CoV-2 enzyme targets. These studies will not be discussed further here.

**Figure 2:**
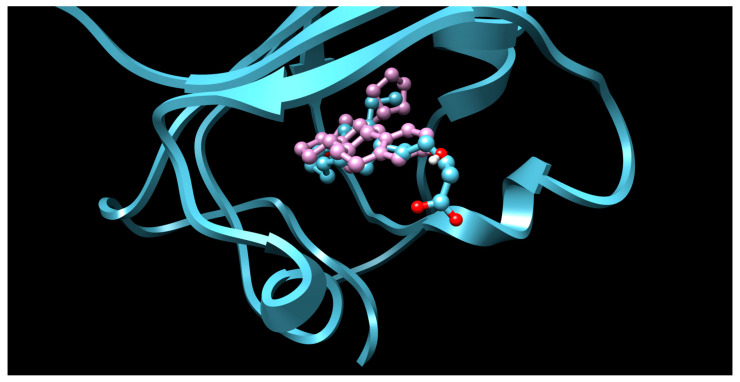
Superimposition of estradiol (pink) with linoleate (cyan) in the spike FABP.

**Figure 3:**
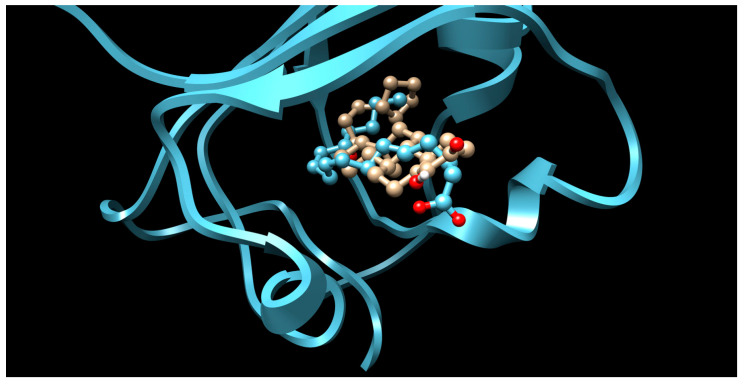
Superimposition of oxabalone (brown) with linoleate (cyan) in the spike FABP.

**Figure 4:**
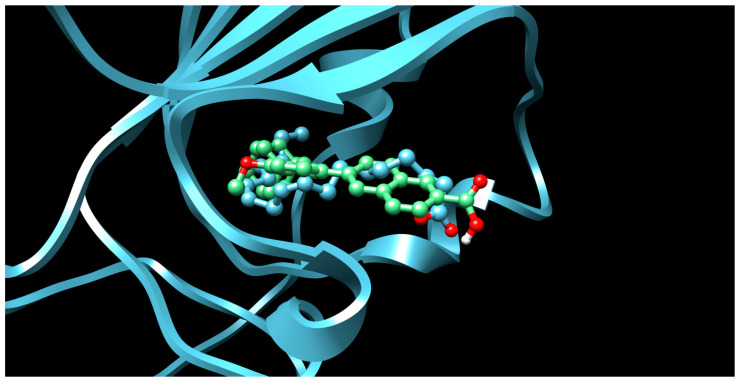
Superimposition of adapalene (green) on linoleate (cyan) in the FABP of spike.

There were 17 compounds in common with those reported by Shoemark et al [3]., and many of our predicted drugs are unique to our study. Notably, we screened a larger number of compounds than they did, which may explain the differences. However, there was a good correlation between the binding energies calculated by Shoemark et al. and by our study, with an r^2^ value > 0.70. Figure 2, Figure 3 and Figure 4 show the binding poses of three of the top ligands superimposed with linoleic acid in the FABP.

**Figure 5:**
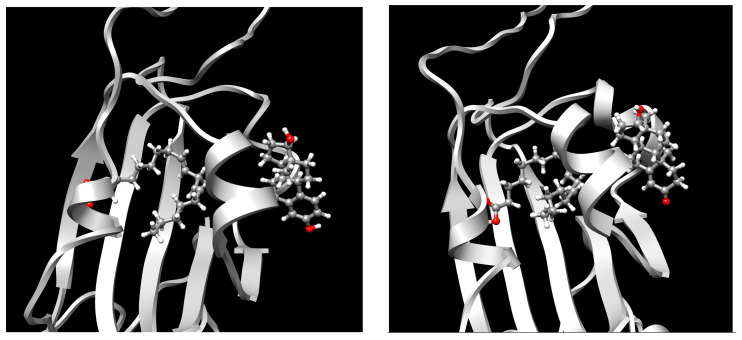
Cooperative binding of testosterone (**left**) and estrogen (**right**) with FABP in the presence of bound LA (overlaying beta strands). Hormones are to the right of the right-hand spike helix.

We also observed that, even with LA in the FABP, both testosterone and estrogen could bind to the pocket, albeit near the pocket entrance. Figure 5 shows the binding orientation of these sex hormones when the FABP is bound to LA. We calculated the binding energies of LA + testosterone as −38.54 kcal/mol and LA + estrogen as −44.54 kcal/mol. The binding energy of LA to the spike alone is −22.17 kcal/mol, while that of testosterone is −19.31 kcal/mol and estrogen is −28.57 kcal/mol. Thus, binding of testosterone to the spike + LA contributes 16.4 kcal/mol and estrogen 25.2 kcal/mol to the cooperative binding energy.

This result suggests that synergistic binding of LA (and by implication other fatty acids) with estrogen and testosterone potentially creates more effective locking of the spike in the closed form.

We found no significant univariate or multivariate relationships between log P(octanol/water) (lipophilicity), number of hydrogen bond donors and acceptors, and number of rotatable bonds (flexibility) of FABP ligands and their binding energies (Tables S1 and S2).

## 3. Discussion

The hits in Table 1 comprise multiple drug families. Literature analysis of the modes of action of these classes, and published work on their efficacy against SARS-CoV-2, provide mechanistic information and validate our computational screening approach as useful for identifying repurposing candidates. This approach was adopted in our published repurposing studies on the SARS-CoV-2 main protease (M^pro^), helicase, and RNA-dependent RNA polymerase [8,9,11].

### 3.1. CNS Drugs

Our top repurposing hit was caprospinol, a spirostenol neurosteroid active on the mammalian nervous system through receptors other than steroid hormone nuclear receptors. It has a 22R-hydroxycholesterol structure and is being developed by Samaritan Pharmaceuticals for Alzheimer’s disease. It protects neuronal mitochondria from cytotoxicity and cell death in preclinical studies by binding to beta-amyloid peptide and preventing its oligomerization and entry into neurons [12]. It does not bind to steroid receptors and should be screened for SARS-CoV-2 activity, as no data are currently available. Metixene, an anticholinergic antiparkinsonian agent, reduced infectivity of SARS-CoV-2 in 293T-ACE2 and Vero E6 cells by 50% below 1 µM with a selectivity index >10 [13]. The antipsychotic drug, aripiprazole, inhibits SARS-CoV-2 entry into ACE2-expressing HEK293T cells by 82% [14]. Villoutriex and co-workers reported that it inhibited SARS-CoV-2 in Vero E6 cells with an IC_50_ of 13 µM [15]. Another top hit was setiptiline, a tetracyclic antidepressant that acts as a noradrenergic and specific serotonergic antidepressant. No in vitro or in vivo SARS-CoV-2 data are available. Lurasidone is an antipsychotic drug for acute depression and schizophrenia, predicted to bind to the S protein RBD and other molecular targets [16]. In vitro assays in Huh7 cells showed lurasidone inhibited SARS-CoV-2 with an EC_50_ of 18.0 ± 4.6 µM [17]. The antipsychotic, penfluridol, inhibited SARS-CoV-2 replication in Vero E6 cells with an IC_50_ of 2.4 µM and CC_50_ of 12.9 µM [18]. Fluspirilene inhibited SARS-CoV-2 infection in Vero E6 cells with an EC_50_ of 3.2 µM and CC_50_ of 30 µM [19]. In vitro activity was also reported to be EC_50_ of 5.3 µM and CC_50_ of 30 µM in Vero E6 cells [20], and it inhibited SARS-CoV-1 and MERS-CoV with EC_50_ values of 7.5 µM and 6.0 µM, respectively [21]. Significantly, psychiatric patients on antipsychotics, including fluspirilene, have a lower incidence of COVID-19 disease than expected given their risk factors, which is postulated to be due to antiviral properties [22].

### 3.2. Sex Hormones and Hormone Analogues

Top candidates from our screen have also been reported by others to be potential inhibitors of the spike protein or exhibit in vitro or in vivo activity against SAR-CoV-2 virus. Interestingly, many of the predicted hits are hormones or hormone analogues. Oxabolone, one of the strongest predicted binders, is a synthetic anabolic-androgenic steroid of the nandrolone (19-nortestosterone) group that includes nandrolone phenpropionate and nandrolone decanoate and is predicted to have tight binding. Men with severe COVID-19 have been shown to have a significantly lower testosterone level compared to those with mild COVID-19 or uninfected patients [23]. Notably, male sex, age, comorbidities, and smoking habits were shown to be risk factors for death from COVID-19 [24]. However, estrogens are protective against severe COVID-19. Reduced testosterone was correlated with a poor prognosis [25]. Androstenedione, an endogenous precursor of testosterone and other androgens and estrogens, was also predicted to be a good FABP ligand. As well as its endogenous prohormone function, androstenedione is also a weak androgen and estrogen like other dehydroepiandrosterone metabolites. Interestingly, several nortestosterone and estradiol compounds also had a high binding score. Figure 2 and Figure 3 show the superimposition of estradiol and oxabolone with linoleate in the FABP.

Hence, estrogen and testosterone may bind competitively in the FABP, with estrogen being more effective in locking the S protein in the closed conformation. Estradiol benzoate inhibits the interaction between S and ACE2 with an IC_50_ of 17.5µM [26]. Yang et al. also reported that it inhibited viral entry by targeting the six-helix (6-HB) fusion core of SARS-CoV-2 S protein. It inhibited SARS-CoV-2 invasion into cells with an EC_50_ of 270 nM, and infection by SARS-CoV-2 in Vero-E6 cells with an EC_50_ of 6.75µM [27]. A clinical trial is assessing estradiol cypionate 5 mg/mL and progesterone 200 mg oral capsule for COVID-19 treatment (NCT04865029). Conspicuously, the antidiabetic agent troglitazole has been shown to reduce testosterone levels in men [28] and was also predicted to be a good binder to the FABP by our MD simulations. This raises the question as to whether this, and related drugs, may be useful for treating COVID-19 by inhibiting the S protein, despite reducing testosterone.

### 3.3. Corticosteroids

Another steroid hormone hit was hydrocortisone, a natural corticosteroid with glucocorticoid and mineralocorticoid activity. Glucocorticoids were the first recommended treatment for severe COVID-19 infection. The REMAP-CAP clinical trial demonstrated that intravenous hydrocortisone improved recovery and reduced COVID-19 mortality, presumably due to its anti-inflammatory action. It is interesting to speculate whether binding to the FABP may have contributed this outcome, which was similar to monoclonal antibodies, tocilizumab, and sarlumab [29]. Cortisone acetate, a cortisone prodrug, was also predicted to bind to the FABP. 

5-α-pregnane-3-β-ol hemisuccinate, another anti-inflammatory glucocorticoid, also exhibits relatively strong binding to the FABP. Pregnenolone, a precursor to cortisol, progesterone, dehydroepiandrosterone, estrogen, and testosterone, is yet another natural steroid hormone predicted to bind the FABP [30]. As a commonly used hormone supplement, pregnenolone should be screened for direct SARS-CoV-2 activity. Altrenogest, a progestin of the 19-nortestosterone group widely used in veterinary medicine to suppress or synchronize estrus, was another steroid hormone that bound tightly to the FABP. Ouabain, a plant derived cardiac glycoside, inhibited the SARS-CoV-2 strain USA-WA1/2020 in Vero E6 cells with an EC_50_ of 45 nM [31].

### 3.4. Vitamin A, D, K, and Analogues

Another hormone in Table 1 is calcitriol (1,25-dihydroxycholecalciferol vitamin D), a steroid hormone that binds to and activates the vitamin D receptor in the nucleus of the cell, regulating multiple genes. Other groups have identified calcitriol and other steroids as ligands for the S protein FABP [6,32]. Unlike other steroids, calcitriol interacts with Arg408 and Gln409 on S, which increases its binding affinity. It was recently reported that post-infection treatment with 10 μM calcitriol resulted in a 20-fold reduction of SARS-CoV-2 titre in Vero E6 cells [33]. Notably, calcitriol also produced a 5-fold reduction in virus titre in the primary human nasal epithelial cell lines, known to be a target of SARS-CoV-2 in vivo. Interestingly, vitamin D levels have also been shown to be negatively correlated with morbidity and mortality of COVID-19 cases [34], which is consistent with calcitriol binding to the FABP in vivo, keeping the spike in the inactive, closed conformation. Inecalcitol, an analogue of calcitriol and a vitamin D3 receptor (VDR) agonist, also had a strong FABP binding. Another highly ranked drug involved in the calcium signalling pathway is cinacalcet, with a predicted binding energy of −33.02 kcal/mol. It mimics the action of calcium by allosteric activation of the calcium-sensing receptor and is used to treat hypercalcaemia due to hyperparathyroidism. It exhibits strong activity against SARS-CoV-2 of 3.0 ± 0.4 µM in Vero E6 cells with a 7-fold selectivity index (SI) [35]. Adapalene, a third-generation topical retinoid, exhibited a strong binding affinity to the mutant S protein of Omicron variant in docking and MD studies [36]. It also had an EC_50_ value of 9.6 µM in Vero E6 cells, which was attributed to binding to the spike RBD [37]. Shoemark and colleagues also reported Vitamin K2 as a best binder, and adapalene and vitamin A as a tight binders to the FABP [6]. Figure 4 shows a superimposition of linoleic acid with adapalene in the spike FABP.

Ligand interaction with the FABP is the first critical step in generation of the locked form of spike glycoprotein with a much lower affinity for ACE2 than the open form. Interestingly, both vitamin K and vitamin D deficiency were independently associated with higher COVID-19 disease severity. Menatetrenone, one of the nine forms of vitamin K2, also exhibited strong binding to the FABP in our studies [38].

### 3.5. Cancer Drugs

Golvatinib, another top repurposing hit, was shown by us and others to potentially inhibit the main protease [8]. It exhibits modest in vitro activity against SARS-CoV-2 in Vero E6 cells at 800 nM [39]. Irinotecan, a chemotherapeutic drug, was shown to completely block the interaction of ACE2 with the spike using a surface plasmon resonance competition assay (K_D_ = 800 nM) but was toxic to Vero-E6 cells [40]. Docking and MD studies also identified irinotecan as a tight binder to triple-mutated viral S1 spike proteins of SARS-CoV-2 [41]. The antidiabetic drugs gliquidone, reglitazar, and englitazone also bound strongly to the FABP. Manickavasagam reported MD calculations that suggested gliquidone bound to a similar region to the FA binding site (residues 330–480) with good affinity [42]. Sorafenib exhibited an IC_50_ of 1.6 µM and CC_50_ of 1.2 µM against a live SARS-CoV-2 clinical isolate in Caco-2 cells [43]. Romeo et al. found that laniquidar bound strongly to S, consistent with our modelling studies on the FABP [44]. The Abelson murine leukemia viral oncogene homolog 1 (Abl) kinase inhibitor, nilotinib, inhibited SARS-CoV-2 in Vero-E6 cells and Calu-3 cells with EC_50_ values of 1.44 μM and 3.06 μM, respectively [45]. It was predicted to bind to two cryptic pockets in S and potentially stabilize intermediate structures and prevent formation of spike conformations responsible for viral entry [46]. Nilotinib inhibited spike-ACE2 binding with an IC_50_ of 4 µM [26]. Ponatinib, another Abl kinase inhibitor, blocked SARS-CoV-2 in Huh7 cells with an EC_50_ of 1.1 µM and CC_50_ of 9 µM [17]. Brequinar, another antineoplastic agent, displayed in vitro activity of 300 nM against SARS-CoV-2 in Vero CCL-81 [47] and Vero E6 cells [48]. It is being assessed in a phase I randomized clinical trial (NCT04425252) of COVID-19 patients.

### 3.6. Other Drug Classes

Indacaterol is an ultra-long-acting beta-adrenoceptor agonist for the treatment of chronic obstructive pulmonary disease (COPD). Awad et al., using a virtual screening approach, suggested that indacaterol binds stably near the C terminal domain of the S1 subunit (CTD1) [49]. Another beta-adrenoceptor agonist, vibegron, also exhibits high predicted binding to the FABP. No in vitro or in vivo activity data on indacaterol or vibegron are available, making them interesting candidates to test.

Lazniewski et al. conducted a comprehensive MD study of the interaction of pranlukast, a bronchospasm antagonist, and several other repurposed drugs with the spike protein from several strains of SARS-CoV-2 [50]. They found that pranlukast stays inside the RBD of the delta lineage for only around 50 ns, then drifts away and binds to a neighbouring cavity adjacent to the FABP residues. Singh and Dhar also reported computational evidence that pranlukast binds to the spike [51], as did Shoemark et al. However, Imamura and co-workers reported that pranlukast showed no activity against SARS-CoV-2 in Vero E6 cells [52]. Behaviour similar to pranlukast was reported for siponimod by MD simulations [53]. Binding to the second site of the FFABP was predicted to be stronger then binding to the ACE2 RBD.

The ACE2 receptor-blocking antihypertensive, telmisartan, was reported to reduce day 14/15 COVID-19 in obese patients by 72% [54] by disrupting the binding of SARS-CoV-2 to ACE2. There are clinical trials for COVID-19 in elderly patients (NCT04356495 and NCT04355936) that reported good efficacy, reducing the time to discharge by half [55]. It is a relatively potent lead molecule with an in vitro EC_50_ of 1.0 µM in Vero E6 cells against SARS-CoV-2/Human/IND/CAD1339/2020 [56]. As we found that it bound well to the FABP, we speculate whether its clinical effectiveness is due to blocking the conformational switch required for spike to bind to ACE2. Manidipine, a calcium channel blocker antihypertensive, inhibits SARS-CoV-2 infection in Huh7 and Vero E6 cells with IC_50_ values of 2 µM and 7.5 µM, respectively [57]. Reported in vitro activity in HeLa-ACE2 and Calu-3 cell lines was 6.9 µM (CC_50_ > 17) and 5.5 µM (CC_50_ > 30) [58]. Like lumacaftor, it was also predicted by MD simulations to form strong hydrogen bonds with K417 in the FABP [50].

The protein-folding chaperone cystic fibrosis drug, lumacaftor, was reported to inhibit viral replication in a Vero E6 cell-based SARS-CoV-2 infection assay with an IC_50_ of 84 µM and CC_50_ of 315 µM and may be a repurposed therapeutic [40]. Tezza et al. used MD simulations to show at least one key hydrogen bonding interaction with K417, and hydrophobic residues of the FABP [59].

The HIV antiviral agent tipranavir can achieve sufficient tissue concentration in vivo to be effective at its EC_50_ value of 8 µM [60]. It was predicted to be an inhibitor of the S RBD by in silico docking studies reported by several researchers [61].

Atovaquone (Mepron), a synthetic hydroxy-naphthoquinone antiparasitic, is in clinical trials for COVID-19 treatment (NCT04456153 and NCT4339426). It inhibits replication of the Frieburg strain of SARS-CoV-2 in Vero E6 cells with an IC_50_ of 2.7 µM and produced a 10^5–^10^6^-fold reduction in viral progeny with low toxicity. Importantly, it exhibited an IC_50_ of 30 µM in human lung epithelial Calu-3 cells, reducing the virus by 10^4^-fold, with low toxicity [62]. Conspicuously, they showed that atovaquone potently inhibited replication of SARS-CoV-2 alpha, beta, and delta variants and retained full antiviral activity in a primary human airway epithelium cell culture model. Ahmed et al. also determined the IC_50_ for atovaquone for SARS-CoV-2 as 1.5µM in Vero E6 cells and 6.6 µM in Huh7.5 cells. It distributed effectively to lungs and epithelial lining fluid [63].

As the SARS-CoV-2 virus continues to mutate its RBD, enabling it to escape neutralising antibodies, other more conserved domains within the spike protein may serve as long-lasting and more reliable targets for inhibiting virus infectivity. As shown here, the FABP in the S protein is not just conserved across SARS-CoV-2 variants but is also a feature of the SARS and MERS spike proteins, suggesting that it plays a critical role in virus infectivity. Most likely, its role is to maintain the spike protein in the closed conformation [64] until such time as the virus is ready to bind ACE2, shielding the ACE2 binding domain in the RBD from recognition and neutralisation by anti-RBD antibodies. It undergoes a conformation change in spike protein potentially triggered by interactions with the stalk or N-terminal domains, or to a RGD motif in the RBD [64].

Shoemark et al. similarly identified vitamins, retinoids, and steroids as potential ligands for the FABP, focusing on vitamin K and dexamethasone. They further postulated that the FABP represents a dynamic responsive element that provides an evolutionary advantage, allowing a temporary escape from neutralizing immunoglobulin G during peak inflammatory phases by coupling the RBD open-to-close equilibrium to the abundance of FAs. This model ignores the potential for extracellular ligands such as hormones with an even higher affinity than FFAs, by displacing linoleic acid from the FABP, to maintain the S protein in a closed conformation, or by displacing LA to trigger the RBD to open, or a combination of these.

Our MD simulations with multiple ligands demonstrated that it is possible for more than one ligand to bind in the pocket at the same time, and this may then help explain phenomena such as the protective effect of oestrogen and high levels of testosterone.

Cortisol may play an important role in regulating SARS-CoV-2 infectivity by binding to the spike protein and regulating its function [65]. Moreover, cortisol concentration has been shown to dependently inhibit the interaction between ACE2 and the Beta variant, containing mutations E484K, K417N, and N501Y [66]. It is interesting to speculate that the major diurnal rhythm of serum cortisol may similarly entrain a diurnal rhythm in the proportion of S protein that is open or shut, and thereby the time of maximal viral infectivity, as cortisol levels are high during the morning.

Cortisol and dexamethasone facilitate the heat-induced unfolding of SARS-CoV-2 S1, suggesting that they counteract the effect of LA and induce opening of the RBD. This would imply that high morning cortisol may induce maximum SARS-CoV-2 infectivity when the immune system is maximally suppressed by the morning peak in cortisol. Hence, the virus could synchronise its activity to exploit diurnal patterns in immune responsiveness, something we initially described more than 25 years ago.

Paradoxically, cortisol and dexamethasone were shown to inhibit S1 binding to ACE2, but it is likely that this occurs at higher than physiological, i.e., therapeutic concentrations. This could then explain why therapeutic levels of glucocorticoids reduce disease severity, an effect that may also reflect glucocorticoid suppression of inflammation rather than its effects on S protein. Interestingly, specific mutations in Delta and Omicron variants may impact the binding of glucocorticoids to S1 and hence may also affect glucocorticoid inhibition of S1-ACE2 interactions [66].

This is consistent with recent studies on diurnal variation in positive SARS-CoV-2 PCR tests, which peak around 1400 h and had a 1.7-fold variation over the day after adjustment for age, sex, race, testing location, month, day of week, and lower cycle threshold values during the day for positive samples [67]. Recent in vitro data also identified circadian effects on SARS-CoV-2, SARS, and alpha NL63 infection and replication in lung epithelial cells [68]. Notably, many other hormones predicted to bind spike FABP, such as oestrogen, also have diurnal patterns of release, suggesting a complex and constantly changing relationship between the hormone environment in vivo and the infectivity of the virus.

This study provides novel insights into the SARS-CoV-2 S protein and its potential regulation by endogenous hormones and drugs. Many of the candidate drugs have yet to be tested for activity against the virus, and there is now a strong rationale for doing so. Much remains to be learned about the structure of the spike trimer, how its RBD opens and closes, and what controls it.

## 4. Materials and Methods

Overall, 11,875 FDA-approved drugs in sdf format were downloaded from Drugbank. The drugs were downloaded in sdf format and converted to pdbqt format. Raccoon was used to convert 3D coordinates of each compound. LA bound SARS-CoV-2 spike RBD receptor (PDB ID–6ZB5) was used for docking study. The grid size used was set to 1Å, the maximum number of binding modes to output was fixed at 10, and the exhaustiveness level was set to 8. LA was removed using UCSF Chimera prior to the docking. Autodock Vina was used to virtually screen using an Oracle cloud-computing platform. Docked structures were analyzed by UCSF Chimera and LigPlot+ software to illustrate hydrogen-bond and hydrophobic interactions.

The 100 drugs with the most favorable binding energies were selected for further analysis. Subsequent MD simulations used Gromacs2019.3 (http://www.gromacs.org/, accessed on 24 May 2022) under the CHARMM forcefield, NPT ensemble at 310 K, using periodic boundary conditions. The topology files of the drugs were prepared using Swissparam (http://www.swissparam.ch/, accessed on 24 May 2022). The simulation box was filled with SPC water molecules and 150 mM NaCl ions to neutralize the charge. The complexes were subjected to 2500 cycles of steepest descent minimization followed by 5000 cycles of conjugate gradient minimization. Berendsen barostat and V-rescale thermostat was used to control the pressure and temperature. Finally, a 20 ns production run was performed. During the MD procedure, the SHAKE algorithm was used to constrain all covalent bonds involving hydrogen atoms. The time step was set to 2 fs. The structural stability of the complex was monitored by the RMSD and RMSF values of the backbone atoms of the entire protein. Calculations were also performed for up to 100 ns on several drugs to ensure that 20 ns was sufficiently long for convergence.

MM/PBSA calculations were conducted by GMXPBSA2.1, a suite of Bash/Perl scripts for streamlining MM/PBSA calculations on structural ensembles derived from Gromacs trajectories. In the current study, we used 100 frames at equal distances from 20 ns trajectory files. The estimated uncertainties in the binding energies were <1 kcal/mol.

Molecular mechanics Poisson–Boltzmann surface area (MM/PBSA) and molecular mechanics generalized Born surface area (MM/GBSA) are very popular methods for binding free energy prediction, since they are more accurate than most scoring functions of molecular docking and less computationally demanding than alchemical free energy methods. MM/PBSA and MM/GBSA have been widely used in biomolecular studies such as protein folding, protein–ligand binding, protein–protein interactions, etc. A recent comprehensive review of MM/PB(GB)SA binding energy calculations suggested that, in practice, the free energies may not have absolute accuracy because of inadequate conformational sampling, but the relative free energies measured by r^2^ are still satisfactory [69]. This reinforces conclusions in an earlier paper by Hou et al. [69]. Other reports suggest that MM/PBSA binding energies were better for ranking than docking scoring functions [70], while others have shown no significant difference [71], with both being less effective than MD alchemical methods. Our prior studies on repurposing drugs active against SARS-CoV-2 enzyme targets also established a strong correlation between MM/PBSA energies and those calculated from a thermodynamic cycle [9,11]. A critical review of simulation methods to study SARS-CoV-2 and repurpose drugs has been published recently by Muratov et al. [72].

## Figures and Tables

**Table 1:** MM/PBSA binding energies G (kcal/mol) of top 100 docking candidates. Estimated uncertainties in the binding energies were <1 kcal/mol.

Drug	ΔG	Drug	ΔG	Drug	ΔG	Drug	ΔG
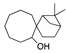 Caprospinol	−38.89	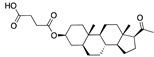 5-α-pregnane−3-β-ol hemisuccinate	−29.60	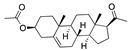 Pregnenolone acetate	−27.75	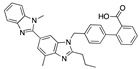 Telmisartan	−23.88
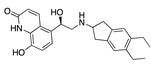 Indacaterol	−35.99	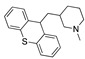 Metixene	−29.50	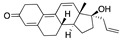 Altrenogest	−27.58	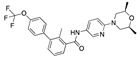 Sonidegib	−23.72
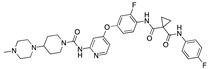 Golvatinib	−35.37	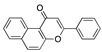 β-Naphthoflavone	−29.32	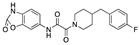 Radiprodil	−27.40	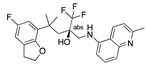 Mapracorat	−23.29
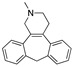 Setiptiline	−35.04	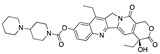 Irinotecan	−29.24	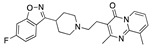 Ocaperidone	−27.36	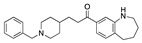 Zanapezil	−23.16
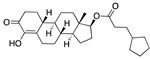 Oxabolone cypionate	−34.78	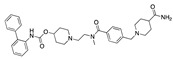 Revefenacin	−29.16	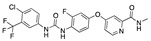 Regorafenib	−27.31	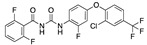 Flufenoxuron	−22.92
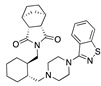 Lurasidone	−33.04	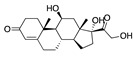 Hydroxycortisone (Cetacort)	−29.16	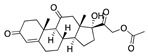 Cortisone acetate	−27.16	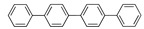 p-quaterphenyl	−22.62
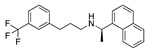 Cinacalcet	−33.02	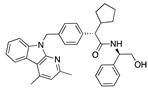 Implitapide	−29.11	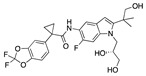 Tezacaftor	−26.85	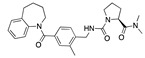 Fedovapagon	−22.44
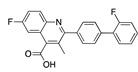 Brequinar	−32.94	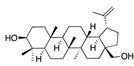 Betulin	−28.83	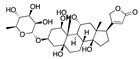 Ouabain	−26.83	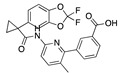 Lumacaftor	−22.33
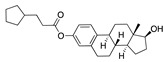 Estradiol cypionate	−32.94	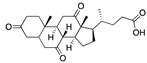 Dehydrocholic acid	−28.82	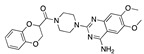 Doxazosin	−26.59	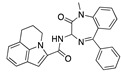 Tarazepide	−21.94
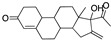 Segesterone	−32.80	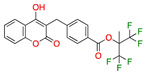 Tecarfarin	−28.80	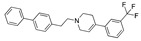 Paliroden	−26.56	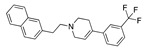 Xaliproden	−21.92
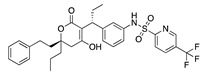 Tipranavir	−32.39	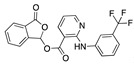 Talniflumate	−28.56	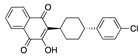 Mepron (atovaquone)	−26.35	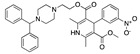 Manidipine	−21.63
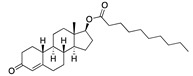 Nandrolone decanoate	−32.33	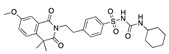 Gliquidone	−28.54	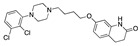 Aripiprazole	-25.96	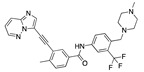 Ponatinib	−21.19
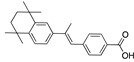 Arotinoid acid	−32.05	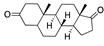 Androstanedione	−28.54	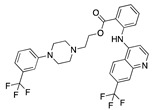 Antrafenine	−25.89	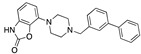 Bifeprunox	−21.16
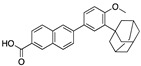 Adapalene	−32.03	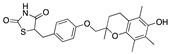 Troglitazone	−28.53	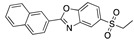 Ezutromid	−25.86	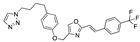 Mubritinib	−20.81
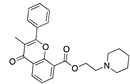 Flavoxate 4.3 0 5 6	−31.70	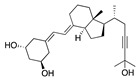 Inecalcitol	−28.26	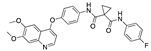 Cabozantinib	−25.81	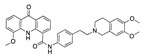 Elacridar	−20.28
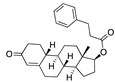 Nandrolone phenpropionate	−31.52	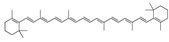 β-carotene	−28.26	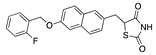 Netoglitazone	−25.53	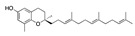 Tocotrienol	−20.11
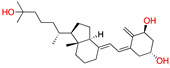 Calcitriol	−31.06	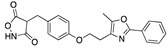 Reglitazar	−28.25	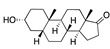 Etiocholanolone	−25.44	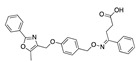 Imiglitazar	−20.09
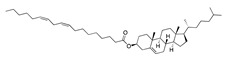 Cholesteryl linoleate	−30.98	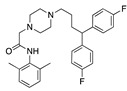 Lidoflazine	−28.11	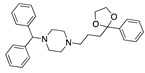 Dotarizine	−25.16	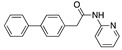 Difenpiramide	−19.56
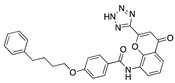 Pranlukast	−30.79	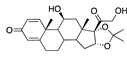 Desonide	−28.08	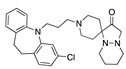 Mosapramine	−24.87	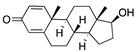 1-Testosterone	−19.31
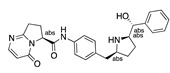 Vibegron	−30.72	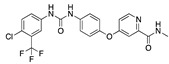 Sorafenib	−28.04	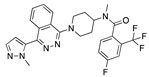 Taladegib	−24.73	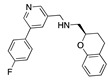 Sarizotan	−18.99
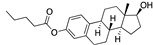 Estradiol valerate	−30.60	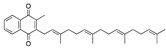 Menatetrenone	−27.98	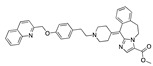 Laniquidar	−24.72	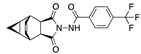 Tecovirimat	−17.77
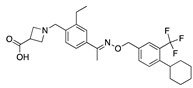 Siponimod	−30.58	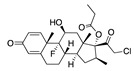 Clobetasol propionate	−27.95	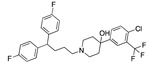 Penfluridol	−24.67	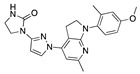 Emicerfont	−17.57
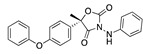 (S)-famoxadone	−30.11	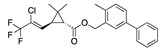 Bifenthrin	−27.78	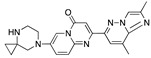 Risdiplam	−24.05	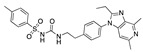 Grapiprant	−17.33
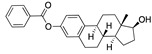 Estradiol benzoate	−29.87	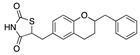 Englitazone	−27.75	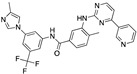 Nilotinib	−24.03	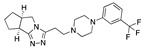 Lorpiprazole	−15.96
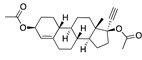 Ethynodiol diacetate	−29.75	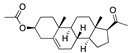 Pregnenolone acetate	−27.75	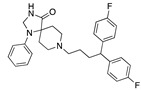 Fluspirilene	−23.95	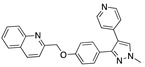 Mardepodect	−15.31

## Data Availability

Data are available from the La Trobe University data archive OPAL (Figshare). DOI 10.26181/22114622.

## Supplementary Materials

The following supporting information can be downloaded at: https://www.mdpi.com/article/10.3390/ijms24044192/s1.

## References

[1] Pushpakom S., Iorio F., Eyers P.A., Escott K.J., Hopper S., Wells A., Doig A., Guilliams T., Latimer J., McNamee C. (2019). Drug repurposing: Progress, challenges and recommendations. Nat. Rev. Drug Disc..

[2] Du L., He Y., Zhou Y., Liu S., Zheng B.J., Jiang S. (2009). The spike protein of SARS-CoV--a target for vaccine and therapeutic development. Nat. Rev. Microbiol..

[3] Toelzer C., Gupta K., Yadav S.K.N., Borucu U., Davidson A.D., Kavanagh Williamson M., Shoemark D.K., Garzoni F., Staufer O., Milligan R. (2020). Free fatty acid binding pocket in the locked structure of SARS-CoV-2 spike protein. Science.

[4] Goc A., Niedzwiecki A., Rath M. (2021). Polyunsaturated omega-3 fatty acids inhibit ACE2-controlled SARS-CoV-2 binding and cellular entry. Sci. Rep..

[5] Deganutti G., Prischi F., Reynolds C.A. (2021). Supervised molecular dynamics for exploring the druggability of the SARS-CoV-2 spike protein. J. Comput. Aided Mol. Des..

[6] Shoemark D.K., Colenso C.K., Toelzer C., Gupta K., Sessions R.B., Davidson A.D., Berger I., Schaffitzel C., Spencer J., Mulholland A.J. (2021). Molecular simulations suggest vitamins, retinoids and steroids as ligands of the free fatty acid pocket of the SARS-CoV-2 spike protein. Angew. Chem..

[7] Marion I.U., Marion A. (2020). Molecular modelling reveals eight novel druggable binding sites in SARS-CoV-2′s spike protein. ChemRxiv.

[8] Piplani S., Singh P., Petrovsky N., Winkler D.A. (2022). Computational repurposing of drugs and natural products Against SARS-CoV-2 main protease (M(pro)) as potential COVID-19 therapies. Front. Mol. Biosci..

[9] Piplani S., Singh P., Winkler D.A., Petrovsky N. (2022). Potential COVID-19 Therapies from computational repurposing of drugs and natural products against the SARS-CoV-2 helicase. Int. J. Mol. Sci..

[10] Piplani S., Singh P.K., Winkler D.A., Petrovsky N. (2021). In silico comparison of SARS-CoV-2 spike protein-ACE2 binding affinities across species and implications for virus origin. Sci. Rep..

[11] Piplani S., Singh P.K., Winkler D.A., Petrovsky N. (2021). Computationally repurposed drugs and natural products against RNA dependent RNA polymerase as potential COVID-19 therapies. Mol. Biomed..

[12] Lecanu L., Tillement L., Rammouz G., Tillement J.P., Greeson J., Papadopoulos V. (2009). Caprospinol: Moving from a neuroactive steroid to a neurotropic drug. Expert Opin. Investig. Drugs.

[13] Le B.L., Andreoletti G., Oskotsky T., Vallejo-Gracia A., Rosales R., Yu K., Kosti I., Leon K.E., Bunis D.G., Li C. (2021). Transcriptomics-based drug repositioning pipeline identifies therapeutic candidates for COVID-19. Sci. Rep..

[14] Lu J., Hou Y., Ge S., Wang X., Wang J., Hu T., Lv Y., He H., Wang C. (2021). Screened antipsychotic drugs inhibit SARS-CoV-2 binding with ACE2 in vitro. Life Sci..

[15] Villoutreix B.O., Krishnamoorthy R., Tamouza R., Leboyer M., Beaune P. (2021). Chemoinformatic analysis of psychotropic and antihistaminic drugs in the light of experimental anti-SARS-CoV-2 activities. Adv. Appl. Bioinform. Chem..

[16] Thurakkal L., Singh S., Roy R., Kar P., Sadhukhan S., Porel M. (2021). An in-silico study on selected organosulfur compounds as potential drugs for SARS-CoV-2 infection via binding multiple drug targets. Chem. Phys. Lett..

[17] Milani M., Donalisio M., Bonotto R.M., Schneider E., Arduino I., Boni F., Lembo D., Marcello A., Mastrangelo E. (2021). Combined in silico and in vitro approaches identified the antipsychotic drug lurasidone and the antiviral drug elbasvir as SARS-CoV2 and HCoV-OC43 inhibitors. Antiviral Res..

[18] Jan J.-T., Cheng T.-J.R., Juang Y.-P., Ma H.-H., Wu Y.-T., Yang W.-B., Cheng C.-W., Chen X., Chou T.-H., Shie J.-J. (2021). Identification of existing pharmaceuticals and herbal medicines as inhibitors of SARS-CoV-2 infection. Proc. Natl. Acad. Sci. USA.

[19] Weston S., Coleman C.M., Haupt R., Logue J., Matthews K., Li Y., Reyes H.M., Weiss S.R., Frieman M.B. (2020). Broad anti-coronavirus activity of food and drug administration-approved drugs against SARS-CoV-2 in vitro and SARS-CoV in vivo. J. Virol..

[20] Xiang R., Yu Z., Wang Y., Wang L., Huo S., Li Y., Liang R., Hao Q., Ying T., Gao Y. (2022). Recent advances in developing small-molecule inhibitors against SARS-CoV-2. Acta Pharmaceut. Sin. B.

[21] Dyall J., Coleman C.M., Hart B.J., Venkataraman T., Holbrook M.R., Kindrachuk J., Johnson R.F., Olinger G.G., Jahrling P.B., Laidlaw M. (2014). Repurposing of clinically developed drugs for treatment of Middle East respiratory syndrome coronavirus infection. Antimicrob. Agents Chemother..

[22] Plaze M., Attali D., Petit A.C., Blatzer M., Simon-Loriere E., Vinckier F., Cachia A., Chrétien F., Gaillard R. (2020). Repurposing of chlorpromazine in COVID-19 treatment: The reCoVery study. Encephale.

[23] Camici M., Zuppi P., Lorenzini P., Scarnecchia L., Pinnetti C., Cicalini S., Nicastri E., Petrosillo N., Palmieri F., D’Offizi G. (2021). Role of testosterone in SARS-CoV-2 infection: A key pathogenic factor and a biomarker for severe pneumonia. Int. J. Infect. Dis..

[24] Wu C., Chen X., Cai Y., Xia J., Zhou X., Xu S., Huang H., Zhang L., Zhou X., Du C. (2020). Risk factors associated with acute respiratory distress syndrome and death in patients with coronavirus disease 2019 pneumonia in Wuhan, China. JAMA Intern. Med..

[25] Rastrelli G., Di Stasi V., Inglese F., Beccaria M., Garuti M., Di Costanzo D., Spreafico F., Greco G.F., Cervi G., Pecoriello A. (2021). Low testosterone levels predict clinical adverse outcomes in SARS-CoV-2 pneumonia patients. Androl..

[26] Tsegay K.B., Adeyemi C.M., Gniffke E.P., Sather D.N., Walker J.K., Smith S.E.P. (2021). A Repurposed drug screen identifies compounds that inhibit the binding of the COVID-19 spike protein to ACE2. Front. Pharmacol..

[27] Yang C., Pan X., Huang Y., Cheng C., Xu X., Wu Y., Xu Y., Shang W., Niu X., Wan Y. (2021). Drug repurposing of itraconazole and estradiol benzoate against COVID-19 by blocking SARS-CoV-2 spike protein-mediated membrane fusion. Adv. Therapeut..

[28] Furnsinn C., Nowotny P., Brunmair B., Gras F., Roden M., Waldhausl W., Vierhapper H. (2002). Thiazolidinediones influence plasma steroids of male obese Zucker rats. Endocrinol..

[29] Angus D.C., Derde L., Al-Beidh F., Annane D., Arabi Y., Beane A., van Bentum-Puijk W., Berry L., Bhimani Z., Bonten M. (2020). Effect of hydrocortisone on mortality and organ support in patients with severe COVID-19: The REMAP-CAP COVID-19 corticosteroid domain randomized clinical trial. JAMA.

[30] Pinna G. (2021). Sex and COVID-19: A protective role for reproductive steroids. Trends Endocrinol. Metab..

[31] Patten J.J., Keiser P.T., Morselli-Gysi D., Menichetti G., Mori H., Donahue C.J., Gan X., Valle I.d., Geoghegan-Barek K., Anantpadma M. (2022). Identification of potent inhibitors of SARS-CoV-2 infection by combined pharmacological evaluation and cellular network prioritization. iScience.

[32] Mansouri A., Kowsar R., Zakariazadeh M., Hakimi H., Miyamoto A. (2022). The impact of calcitriol and estradiol on the SARS-CoV-2 biological activity: A molecular modeling approach. Sci. Rep..

[33] Mok C.K., Ng Y.L., Ahidjo B.A., Hua Lee R.C., Choy Loe M.W., Liu J., Tan K.S., Kaur P., Chng W.J., Wong J.E.-L. (2020). Calcitriol, the active form of vitamin D, is a promising candidate for COVID-19 prophylaxis. bioRxiv.

[34] Ilie P.C., Stefanescu S., Smith L. (2020). The role of vitamin D in the prevention of coronavirus disease 2019 infection and mortality. Aging Clin. Exp. Res..

[35] Li X., Lidsky P.V., Xiao Y., Wu C.T., Garcia-Knight M., Yang J., Nakayama T., Nayak J.V., Jackson P.K., Andino R. (2021). Ethacridine inhibits SARS-CoV-2 by inactivating viral particles. PLoS Pathog..

[36] Fidan O., Mujwar S., Kciuk M. (2022). Discovery of adapalene and dihydrotachysterol as antiviral agents for the Omicron variant of SARS-CoV-2 through computational drug repurposing. Mol. Divers.

[37] Lau E.Y., Negrete O.A., Bennett W.F.D., Bennion B.J., Borucki M., Bourguet F., Epstein A., Franco M., Harmon B., He S. (2021). Discovery of small-molecule inhibitors of SARS-CoV-2 proteins using a computational and experimental pipeline. Front. Mol. Biosci..

[38] Desai A.P., Dirajlal-Fargo S., Durieux J.C., Tribout H., Labbato D., McComsey G.A. (2021). Vitamin K & D deficiencies are independently associated with COVID-19 disease severity. Open Forum. Infect. Dis..

[39] Kaimal J.M., Tampere M., Le T.H., Axelsson U., Xu H., Axelsson H., Bäckström A., Marabita F., Moussaud-Lamodière E., Njenda D. (2022). Subcellular mapping of the protein landscape of SARS-CoV-2 infected cells for target-centric drug repurposing. bioRxiv.

[40] Day C.J., Bailly B., Guillon P., Dirr L., Jen F.E.-C., Spillings B.L., Mak J., von Itzstein M., Haselhorst T., Jennings M.P. (2021). Multidisciplinary approaches identify compounds that bind to human ACE2 or SARS-CoV-2 spike protein as candidates to block SARS-CoV-2–ACE2 receptor interactions. MBio.

[41] Mujwar S. (2021). Computational repurposing of tamibarotene against triple mutant variant of SARS-CoV-2. Comp. Biol. Med..

[42] Manickavasagam P. (2020). Spike protein of SARS-CoV-2: Impact of single amino acid mutation and effect of drug binding to the variant-in silico analysis. Preprints.

[43] Ellinger B., Bojkova D., Zaliani A., Cinatl J., Claussen C., Westhaus S., Reinshagen J., Kuzikov M., Wolf M., Geisslinger G. Identification of inhibitors of SARS-CoV-2 in-vitro cellular toxicity in human (Caco-2) cells using a large scale drug repurposing collection. 2020, Research Square, s.3.rs-23951/v1. 20 April 2020, PREPRINT (Version 1). https://www.researchsquare.com/article/rs-23951/v1.

[44] Romeo A., Iacovelli F., Falconi M. (2020). Targeting the SARS-CoV-2 spike glycoprotein prefusion conformation: Virtual screening and molecular dynamics simulations applied to the identification of potential fusion inhibitors. Virus Res..

[45] Cagno V., Magliocco G., Tapparel C., Daali Y. (2021). The tyrosine kinase inhibitor nilotinib inhibits SARS-CoV-2 in vitro. Basic Clin. Pharmacol. Toxicol..

[46] Dokainish H.M., Re S., Mori T., Kobayashi C., Jung J., Sugita Y. (2022). The inherent flexibility of receptor binding domains in SARS-CoV-2 spike protein. eLife.

[47] Sales-Medina D.F., Ferreira L.R.P., Romera L.M.D., Gonçalves K.R., Guido R.V.C., Courtemanche G., Buckeridge M.S., Durigon É.L., Moraes C.B., Freitas-Junior L.H. (2020). Discovery of clinically approved drugs capable of inhibiting SARS-CoV-2 *in vitro* infection using a phenotypic screening strategy and network-analysis to predict their potential to treat covid-19. bioRxiv.

[48] Xiong R., Zhang L., Li S., Sun Y., Ding M., Wang Y., Zhao Y., Wu Y., Shang W., Jiang X. (2020). Novel and potent inhibitors targeting DHODH are broad-spectrum antivirals against RNA viruses including newly-emerged coronavirus SARS-CoV-2. Protein Cell.

[49] Awad I.E., Abu-Saleh A.A.A., Sharma S., Yadav A., Poirier R.A. (2022). High-throughput virtual screening of drug databanks for potential inhibitors of SARS-CoV-2 spike glycoprotein. J. Biomol. Struct. Dyn..

[50] Lazniewski M., Dermawan D., Hidayat S., Muchtaridi M., Dawson W.K., Plewczynski D. (2022). Drug repurposing for identification of potential spike inhibitors for SARS-CoV-2 using molecular docking and molecular dynamics simulations. Methods.

[51] Singh A., Dhar R. (2021). A large-scale computational screen identifies strong potential inhibitors for disrupting SARS-CoV-2 S-protein and human ACE2 interaction. J. Biomol. Struct. Dynam..

[52] Imamura K., Sakurai Y., Enami T., Shibukawa R., Nishi Y., Ohta A., Shu T., Kawaguchi J., Okada S., Hoenen T. (2021). iPSC screening for drug repurposing identifies anti-RNA virus agents modulating host cell susceptibility. FEBS Open Bio.

[53] Kouznetsova V.L., Zhang A., Miller M.A., Tatineni M., Greenberg J.P., Tsigelny I.F. (2022). Potential SARS-CoV-2 spike protein-ACE2 interface inhibitors: Repurposing FDA-approved drugs. J. Explor. Res. Pharmacol..

[54] Liu D., Wu P., Gu W., Yang C., Yang X., Deng X., Xu J., Jiang J., Jiang C. (2022). Potential of angiotensin II receptor blocker telmisartan in reducing mortality among hospitalized patients with COVID-19 compared with recommended drugs. Cell Discov..

[55] Duarte M., Pelorosso F., Nicolosi L.N., Salgado M.V., Vetulli H., Aquieri A., Azzato F., Castro M., Coyle J., Davolos I. (2021). Telmisartan for treatment of Covid-19 patients: An open multicenter randomized clinical trial. eClinicalMedicine.

[56] Dhaka P., Singh A., Choudhary S., Kumar P., Sharma G.K., Tomar S. (2022). Discovery of anti-SARS-CoV-2 molecules using structure-assisted repurposing approach targeting N-protein. bioRxiv.

[57] Pickard A., Calverley B.C., Chang J., Garva R., Gago S., Lu Y., Kadler K.E. (2021). Discovery of re-purposed drugs that slow SARS-CoV-2 replication in human cells. PLoS Pathog..

[58] Bakowski M.A., Beutler N., Wolff K.C., Kirkpatrick M.G., Chen E., Nguyen T.H., Riva L., Shaabani N., Parren M., Ricketts J. (2021). Drug repurposing screens identify chemical entities for the development of COVID-19 interventions. Nat. Commun..

[59] Trezza A., Iovinelli D., Santucci A., Prischi F., Spiga O. (2020). An integrated drug repurposing strategy for the rapid identification of potential SARS-CoV-2 viral inhibitors. Sci. Rep..

[60] Arshad U., Pertinez H., Box H., Tatham L., Rajoli R.K.R., Curley P., Neary M., Sharp J., Liptrott N.J., Valentijn A. (2020). Prioritization of anti-SARS-Cov-2 drug repurposing opportunities based on plasma and target site concentrations derived from their established human pharmacokinetics. Clin. Pharmacol. Ther..

[61] Behera S.K., Mahapatra N., Tripathy C.S., Pati S. (2021). Drug repurposing for identification of potential inhibitors against SARS-CoV-2 spike receptor-binding domain: An in silico approach. Indian J. Med. Res..

[62] Carter-Timofte M.E., Arulanandam R., Kurmasheva N., Fu K., Laroche G., Taha Z., van der Horst D., Cassin L., van der Sluis R.M., Palermo E. (2021). Antiviral Potential of the antimicrobial drug atovaquone against SARS-CoV-2 and emerging variants of concern. ACS Infect. Dis..

[63] Ahmed M., Farag A., Wang P., Boys I.N., Eitson J.L., Ohlson M.B., Fan W., McDougal M.B., Schoggins J.W., Sadek H. (2021). Identification of Atovaquone and Mebendazole as Repurposed Drugs with Antiviral Activity against SARS-CoV-2.

[64] Staufer O., Gupta K., Hernandez Bücher J.E., Kohler F., Sigl C., Singh G., Vasileiou K., Yagüe Relimpio A., Macher M., Fabritz S. (2022). Synthetic virions reveal fatty acid-coupled adaptive immunogenicity of SARS-CoV-2 spike glycoprotein. Nat. Commun..

[65] Hardy E., Fernandez-Patron C. (2022). Could Endogenous glucocorticoids influence SARS-CoV-2 infectivity?. Cells.

[66] Sarker H., Panigrahi R., Hardy E., Glover J.N.M., Elahi S., Fernandez-Patron C. (2022). Glucocorticoids bind to SARS-CoV-2 S1 at multiple sites causing cooperative inhibition of SARS-CoV-2 S1 interaction with ACE2. Front. Immunol..

[67] McNaughton C.D., Adams N.M., Hirschie Johnson C., Ward M.J., Schmitz J.E., Lasko T.A. (2021). Diurnal variation in SARS-CoV-2 PCR test results: Test accuracy may vary by time of day. J. Biol. Rhythms.

[68] Zhuang X., Tsukuda S., Wrensch F., Wing P.A.C., Schilling M., Harris J.M., Borrmann H., Morgan S.B., Cane J.L., Mailly L. (2021). The circadian clock component BMAL1 regulates SARS-CoV-2 entry and replication in lung epithelial cells. iScience.

[69] Hou T., Wang J., Li Y., Wang W. (2011). Assessing the Performance of the MM/PBSA and MM/GBSA Methods. 1. The accuracy of binding free energy calculations based on molecular dynamics simulations. J. Chem. Inf. Mod..

[70] Breznik M., Ge Y., Bluck J.P., Briem H., Hahn D.F., Christ C.D., Mortier J., Mobley D.L., Meier K. (2023). Prioritizing small sets of molecules for synthesis through in-silico tools: A comparison of common ranking methods. ChemMedChem.

[71] El Khoury L., Santos-Martins D., Sasmal S., Eberhardt J., Bianco G., Ambrosio F.A., Solis-Vasquez L., Koch A., Forli S., Mobley D.L. (2019). Comparison of affinity ranking using AutoDock-GPU and MM-GBSA scores for BACE-1 inhibitors in the D3R Grand Challenge 4. J. Comp.-aided Mol. Des..

[72] Muratov E.N., Amaro R., Andrade C.H., Brown N., Ekins S., Fourches D., Isayev O., Kozakov D., Medina-Franco J.L., Merz K.M. (2021). A critical overview of computational approaches employed for COVID-19 drug discovery. Chem. Soc. Rev..

