# Supplementary material for: Identifying SARS-CoV-2 Drugs Binding to the Spike Fatty Acid Binding Pocket Using In Silico Docking and Molecular Dynamics
Source: Int J Mol Sci. 2023 Feb 20;24(4):4192. doi: 10.3390/ijms24044192 (PMC9966092; doi:10.3390/ijms24044192)
Supplement: Supplementary file 1 [file ijms-24-04192-s001.zip › ijms-2214795-supplementary.pdf]

Identifying SARS-CoV-2 Drugs Binding to the Spike Fatty Acid Binding Pocket Using In Silico Docking and Molecular Dynamics

Supplementary Material

Sakshi Piplani[1-2*], Puneet Singh[1-2*], Nikolai Petrovsky[1-2#] David A. Winkler[3-5#]

[1] College of Medicine and Public Health, Flinders University, Bedford Park 5046, Australia

[2] Vaxine Pty Ltd, 11 Walkley Avenue, Warradale 5046, Australia

[3] Department of Biochemistry and Genetics, La Trobe Institute for Molecular Science, La Trobe University, Melbourne, Victoria, 3086, Australia

[4] Monash Institute of Pharmaceutical Sciences, Monash University, Parkville 3052, Australia

[5] School of Pharmacy, University of Nottingham, Nottingham NG7 2RD. UK

[*] joint first authors

# joint senior authors

Supplementary Information

Table S1. MMPBSA binding energies (kcal/mol) of top 100 docking candidates. Physical properties computed by Cactvs 3.4.8.18

Drug (logP N_{donor} $N_{acceptor}$ $N_{rot\text{-}bonds}$)	ΔG	Drug	ΔG	Drug	ΔG	Drug	ΔG
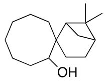<br>**Caprospinol** 8.2 0 4 6	-38.89	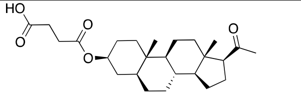<br>**5-α-pregnane-3-β-ol hemisuccinate 4.9 1 5 6**	-29.60	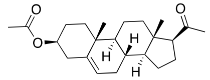<br>**Pregnenolone** acetate 4.8 0 3 3	-27.75	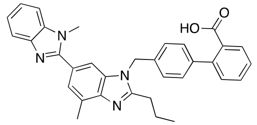<br>**Telmisartan 6.9 1 4 7**	- 23.88
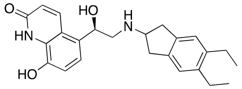<br>**Indacaterol 3.3 4 4 6**	-35.99	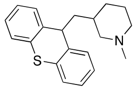<br>**Metixene** 4.9 0 2 2	-29.50	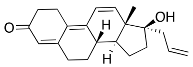<br>**Altrenogest 2.8 1 2 2**	-27.58	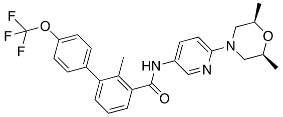<br>Sonidegib 5.8 1 8 5	- 23.72
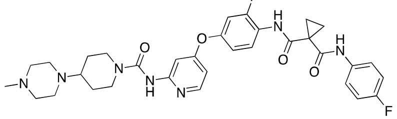<br>**Golvatinib 4.1 3 9 8**	-35.37	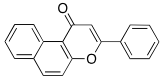<br>β-Naphthoflavone 4.4 0 2 1	-29.32	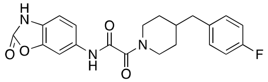<br>Radiprodil 3.0 2 5 3	-27.40	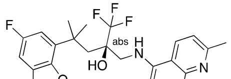<br>Mapracorat 6.2 2 8 6	- 23.29
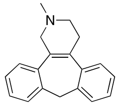<br>**Setiptiline** 3.3 0 1 0	-35.04	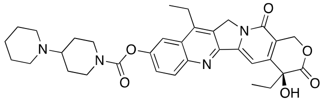<br>**Irinotecan** 3.0 1 8 5	-29.24	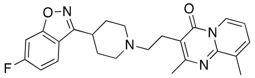<br>Ocaperidone 3.3 0 6 4	-27.36	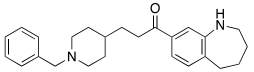<br>Zanapezil 4.9 1 3 6	- 23.16
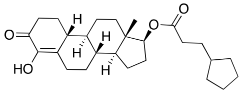<br>**Oxabolone cypionate** 5.7 1 4 5	-34.78	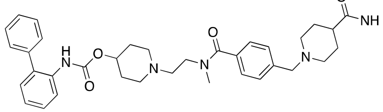<br>Revefenacin 4.1 2 6 11	-29.16	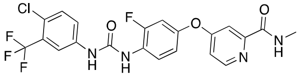<br>Regorafenib 4.2 3 8 5	-27.31	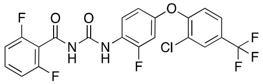<br>Flufenoxuron 6.3 2 9 4	- 22.92
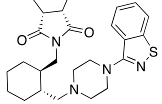<br>**Lurasidone 5.4 0 6 5**	-33.04	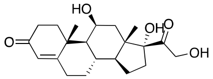<br>**Hydroxycortisone** (Cetacort) 1.6 3 5 2	-29.16	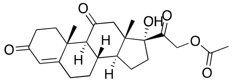<br>**Cortisone acetate 2.1 1 6 4**	-27.16	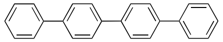<br>p-quaterphenyl 7.3 0 0 3	- 22.62

Drug (logP N_{donor} $N_{acceptor}$ $N_{rot\text{-}bonds}$)	ΔG	Drug	ΔG	Drug	ΔG	Drug	ΔG
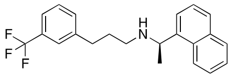<br>**Cinacalcet** 6.1 1 4 6	-33.02	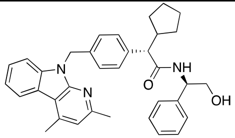<br>Implitapide 7.3 2 3 8	-29.11	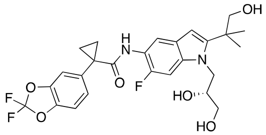<br>Tezacaftor 2.9 4 9 8	-26.85	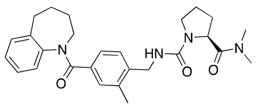<br>Fedovapagon 3.2 1 3 4	- 22.44
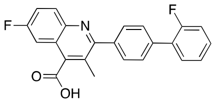<br>**Brequinar 5.6 1 5 3**	-32.94	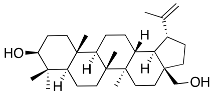<br>Betulin 8.3 2 2 2	-28.83	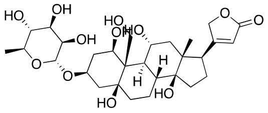<br>**Ouabain** -1.7 8 12 4	-26.83	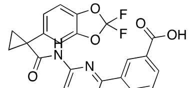<br>**Lumacaftor 4.4 2 8 5**	- 22.33
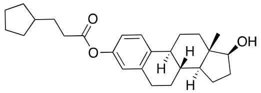<br>**Estradiol cypionate** 7.1 1 3 5	-32.94	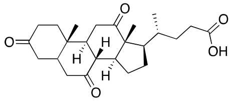<br>Dehydrocholic acid 2.6 1 5 4	-28.82	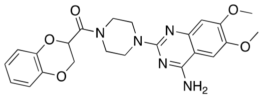<br>Doxazosin 2.5 1 9 4	-26.59	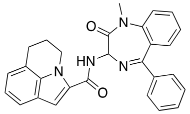<br>Tarazepide 4.6 1 3 3	- 21.94
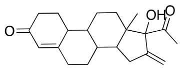<br>Segesterone 2.3 1 3 1	-32.80	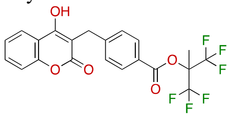<br>Tecarfarin 5.5 1 11 5	-28.80	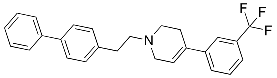<br>Paliroden 6.6 0 4 5	-26.56	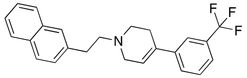<br>Xaliproden 6.2 0 4 4	- 21.92
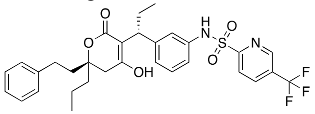<br>**Tipranavir** 7.0 2 10 11	-32.39	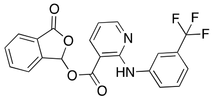<br>Talniflumate 5.3 1 9 5	-28.56	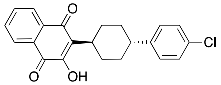<br>**Mepron (atovaquone) 5.2 1 3 2**	-26.35	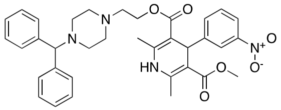<br>**Manidipine 5.6 1 9 11**	- 21.63
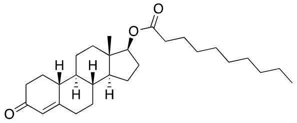<br>**Nandrolone decanoate** 7.3 0 3 10	-32.33	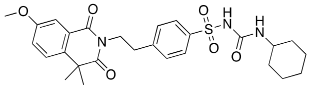<br>**Gliquidone** 4.6 2 6 7	-28.54	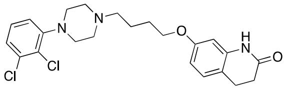<br>**Aripiprazole 4.6 1 4 7**	-25.96	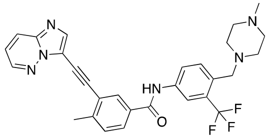<br>**Ponatinib 4.1 1 8 6**	- 21.19
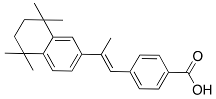<br>Arotinoid acid 7.5 1 2 3	-32.05	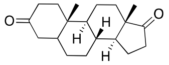<br>**Androstanedione** 3.6 0 2 0	-28.54	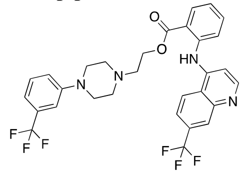	-25.89	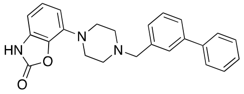<br>Bifeprunox 4.2 1 4 4	- 21.16

Drug (logP N_{donor} $N_{acceptor}$ $N_{rot\text{-}bonds}$)	ΔG	Drug	ΔG	Drug	ΔG	Drug	ΔG
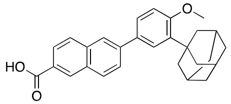 **Adapalene** 7.7 1 3 4	-32.03	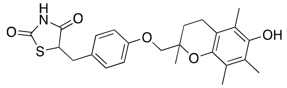 **Troglitazone** 5.1 2 6 5	-28.53	Antrafenine 7.5 1 12 8 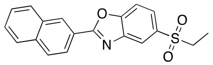 Ezutromid 4.3 0 4 3	-25.86	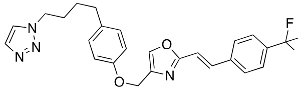 Mubritinib 5.4 0 8 10	- 20.81
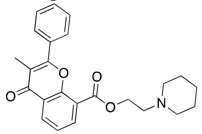 Flavoxate 4.3 0 5 6	-31.70	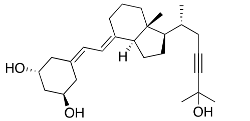 **Inecalcitol** 4.4 3 3 4	-28.26	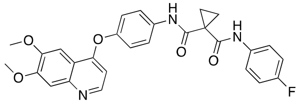 Cabozantinib 5.4 2 7 8	-25.81	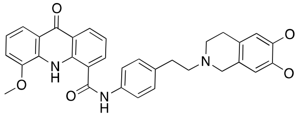 Elacridar 5.6 2 7 8	- 20.28
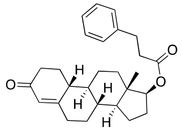 **Nandrolone phenpropionate** 5.1 0 3 5	-31.52	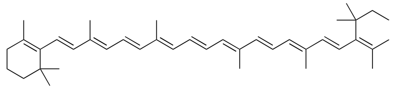 β-carotene 13.5 0 0 10	-28.26	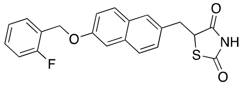 Netoglitazone 4.9 1 5 5	-25.53	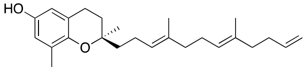 Tocotrienol 8.2 1 2 9	- 20.11
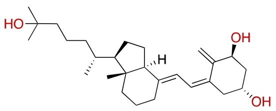 **Calcitriol** 5.1 3 3 6	-31.06	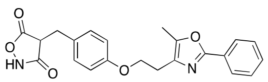 **Reglitazar** 4.0 1 6 7	-28.25	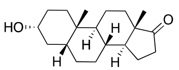 Etiocholanolone 3.7 1 2 0	-25.44	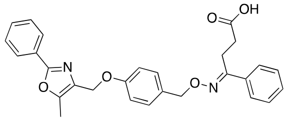 Imiglitazar 5.1 1 7 11	- 20.09
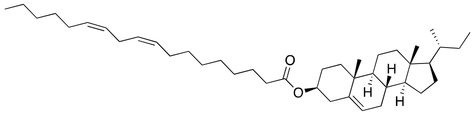 Cholesteryl linoleate 15.9 0 2 21	-30.98	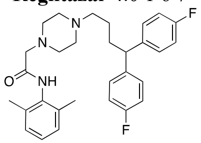 Lidoflazine 6.0 1 5 9	-28.11	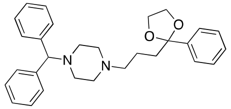 Dotarizine 4.9 0 4 8	-25.16	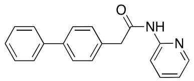 Difenpiramide 3.8 1 2 4	- 19.56
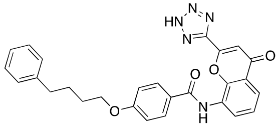 **Pranlukast** 4.2 2 7 9	-30.79	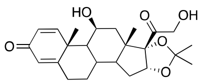 Desonide 2.7 2 6 2	-28.08	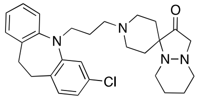 Mosapramine 5.4 1 4 4	-24.87	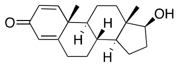 **1-Testosterone 4.0 1 2 0**	- 19.31
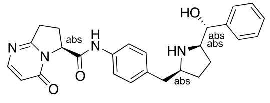 **Vibegron** 1.8 3 5 6	-30.72	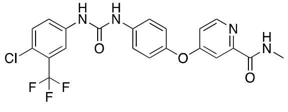 **Sorafenib** 4.1 3 7 5	-28.04	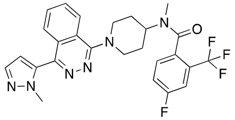	-24.73	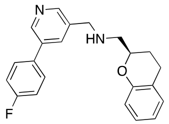	- 18.99

Drug (logP N_{donor} $N_{acceptor}$ $N_{rot\text{-}bonds}$)	ΔG	Drug	ΔG	Drug	ΔG	Drug	ΔG
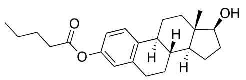 **Estradiol valerate** 6.0 1 3 5	-30.60	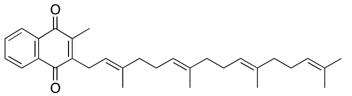 **Menatetrenone** 8.9 0 2 11	-27.98	Taladegib 4.3 0 9 4 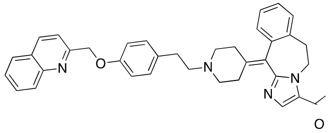 **Laniquidar 5.8 0 6 8**	-24.72	Sarizotan 4.1 1 4 5 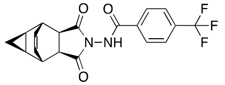 Tecovirimat 2.6 1 6 1	-<br>17.77
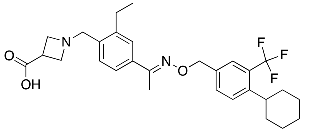 **Siponimod** 4.8 1 8 9	-30.58	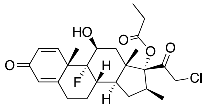 Clobetasol propionate 3.8 1 6 5	-27.95	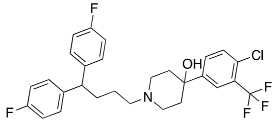 **Penfluridol 7.3 1 7 7**	-24.67	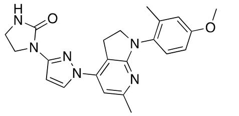 Emicerfont 2.9 1 5 4	-<br>17.57
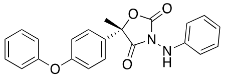 (S)-famoxadone 5.0 1 5 5	-30.11	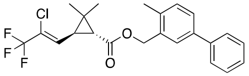 Bifenthrin 6.0 0 5 6	-27.78	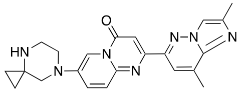 Risdiplam 0.5 1 6 2	-24.05	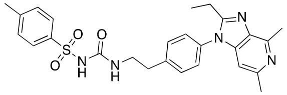 Grapiprant 4.6 2 5 7	-<br>17.33
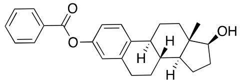 **Estradiol benzoate** 4.5 1 3 3	-29.87	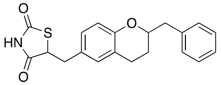 **Englitazone** 4.4 1 4 4	-27.75	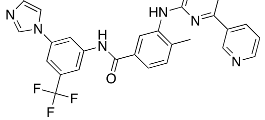 **Nilotinib 4.9 2 9 6**	-24.03	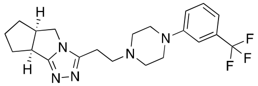 Lorpiprazole 3.3 0 7 4	-<br>15.96
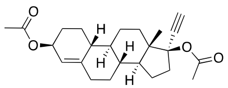 **Ethynodiol diacetate** 3.9 0 4 5	-29.75	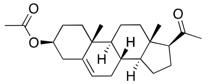 **Pregnenolone acetate** 4.8 0 3 3	-27.75	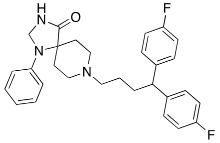 **Fluspirilene 5.6 1 5 7**	-23.95	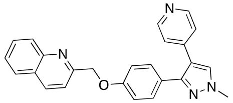 Mardepodect 4.2 0 4 5	-<br>15.31

Table S2. Correlations between physical properties of ligands and binding energies

	logP	N_{donor}	$N_{acceptor}$	N_{rotate}	ΔG
logP	1.00				
N_{donor}	-0.38	1.00			
$N_{acceptor}$	-0.29	0.41	1.00		
N_{rotate}	0.57	0.02	0.23	1.00	
ΔG	-0.14	-0.08	0.12	-0.04	1.00
